# Short-stay crisis units for mental health patients on crisis care pathways: systematic review and meta-analysis

**DOI:** 10.1192/bjo.2022.534

**Published:** 2022-07-25

**Authors:** Katie Anderson, Lucy P. Goldsmith, Jo Lomani, Zena Ali, Geraldine Clarke, Chloe Crowe, Heather Jarman, Sonia Johnson, David McDaid, Paris Pariza, A-La Park, Jared A. Smith, Elizabeth Stovold, Kati Turner, Steve Gillard

**Affiliations:** Division of Nursing, School of Health Sciences, City, University of London, UK; Library Services, St George's, University of London, UK; Improvement Analytics Unit, The Health Foundation, UK; Sunflowers Court, North East London NHS Foundation Trust, UK; Emergency Care, St George's University Hospitals NHS Foundation Trust, London; and Population Health Research Institute, St George's, University of London, UK; NIHR Mental Health Policy Research Unit, Division of Psychiatry, University College London, UK; Care Policy and Evaluation Centre, Department of Health Policy, London School of Economics and Political Science, UK; Collabor8research, London, UK; and Division of Nursing, School of Health Sciences, City, University of London, UK; Population Health Research Institute, St George's, University of London, UK

**Keywords:** Psychiatric nursing, suicide, crisis care, emergency psychiatric care, crisis unit

## Abstract

**Background:**

Internationally, an increasing proportion of emergency department visits are mental health related. Concurrently, psychiatric wards are often occupied above capacity. Healthcare providers have introduced short-stay, hospital-based crisis units offering a therapeutic space for stabilisation, assessment and appropriate referral. Research lags behind roll-out, and a review of the evidence is urgently needed to inform policy and further introduction of similar units.

**Aims:**

This systematic review aims to evaluate the effectiveness of short-stay, hospital-based mental health crisis units.

**Method:**

We searched EMBASE, Medline, CINAHL and PsycINFO up to March 2021. All designs incorporating a control or comparison group were eligible for inclusion, and all effect estimates with a comparison group were extracted and combined meta-analytically where appropriate. We assessed study risk of bias with Risk of Bias in Non-Randomized Studies – of Interventions and Risk of Bias in Randomized Trials.

**Results:**

Data from twelve studies across six countries (Australia, Belgium, Canada, The Netherlands, UK and USA) and 67 505 participants were included. Data indicated that units delivered benefits on many outcomes. Units could reduce psychiatric holds (42% after intervention compared with 49.8% before intervention; difference = 7.8%; *P* < 0.0001) and increase out-patient follow-up care (χ^2^ = 37.42, d.f. = 1; *P* < 0.001). Meta-analysis indicated a significant reduction in length of emergency department stay (by 164.24 min; 95% CI −261.24 to −67.23 min; *P* < 0.001) and number of in-patient admissions (odds ratio 0.55, 95% CI 0.43–0.68; *P* < 0.001).

**Conclusions:**

Short-stay mental health crisis units are effective for reducing emergency department wait times and in-patient admissions. Further research should investigate the impact of units on patient experience, and clinical and social outcomes.

There is an international crisis in acute mental healthcare.^1^ In the USA, the number of emergency department visits for patients with mental health issues increased by a third between 2006 and 2015,^2^ resulting in long waiting times.^3^ A similar picture has emerged in Canada,^4^ the UK^5^ and Australia.^6^ Although a decrease in emergency department presentations was initially reported during the COVID-19 pandemic, numbers have since risen again.^7,8^ This is despite broad consensus that the emergency department is an unsuitable, non-therapeutic environment for people experiencing a crisis in their mental health.^9^ Furthermore, in Europe, the number of in-patient psychiatric beds has decreased.^10^ This adds to pressure for beds and changes the ward environment; typically, levels of distress are higher in those admitted, with the effectiveness of in-patient stays for different patient groups at different levels of distress becoming a topic of serious debate.^11^ People attending the emergency department in crisis and/or admitted for short in-patient stays often experience intense distress, feel suicidal or have attempted suicide.^12–14^ Therefore, there is a need for an appropriate environment to support and assess individuals who feel suicidal. In addition, in-patient admissions can be expensive,^15^ cause harm^16^ and, it has been suggested, are avoidable for about 17% of individuals.^17^ Evidence for the benefits of shorter stays on in-patient wards is inconclusive,^18^ whereas efforts are increasingly being made to improve patient flow in acute and emergency mental health services.^19,20^

Within this context, short-stay crisis units for people in mental health crisis have been developed and introduced.^21^ Variously named emergency psychiatry assessment, treatment and healing units (EmPATH units),^22^ behavioural assessment units,^23^ psychiatric observation units^24^ and psychiatric decision units,^25^ among others, these units are hospital based; allow overnight stays for a short, time-limited period; provide an appropriate environment for stabilisation, assessment and onward referral; and typically aim to reduce emergency department mental health presentations and wait times, and/or psychiatric admissions. An existing systematic review^26^ considered a range of residential alternatives to acute psychiatric admission focusing on crisis hostels, family placements and other forms of community residential services, but did not report on the types of units we consider here. The present review is the first review to focus solely on the effectiveness of hospital-based, short stay crisis units designed to reduce in-patient admissions, emergency department presentations and/or emergency department wait time.

## Method

We conducted a systematic review of quantitative studies of hospital-based mental health crisis units, as described above. The protocol was preregistered on the PROSPERO website (registration number CRD42019151043).^27^ All outcomes with a comparison group were included. The research team was comprised of researchers who bring lived experience of mental distress and using mental health services to their roles, ensuring that experiential knowledge informed the review process,^28^ as well as clinicians and academics. All aspects of the review were co-produced between individuals working from these different perspectives.

### Search strategy

The search followed the PRISMA guidelines.^29^ We searched EMBASE, Medline, CINAHL and PsycINFO databases, using keywords and subject headings from inception to 1 March 2021, supplemented with backward reference searching and forward citation tracking of included studies. We revised our plan to include the Cochrane Central Register of Controlled Trials because of the study types most commonly performed within this area. We included quantitative studies incorporating any comparison (no intervention, a different intervention or within-group comparison) covering a range of designs (single-, double- or triple-blind trials, interrupted time series, quasi-experimental, observational, before-and-after and retrospective studies). Entirely qualitative studies or studies with no comparator were excluded. We did not restrict the search by language. Exemplar papers in the published literature,^23–25^ non-peer reviewed reports and the broad academic, clinical and lived experience of our team were used to coproduce eligibility criteria and search terms. Because of the variability in terminology for the units, we used truncated and adjacent search terms to enhance our search (e.g. (asses* or evaluat* or stabilis* or stabiliz* or crisis or crises or observation*) adj4 (unit or units or facilit* or ward* or room* or suite* or service*)). Full search strategies are available (see Supplementary Fig. 1 available at https://doi.org/10.1192/bjo.2022.534).

### Eligibility criteria

Short-stay crisis units were defined as any mental health assessment service that is (a) hospital-based; (b) allows overnight stay; (c) specifies a short (less than 1 week) length of stay (LOS) and (d) primarily aims to assess and/or stabilise, with the purpose of reducing the need or LOS of standard acute psychiatric admission, and/or reducing mental health presentation or length of wait at the emergency department. Exclusion criteria were non-residential, or community- or non-hospital residential-based assessment or crisis units, and units in which the population were all detained under mental health legislation, all were forensic patients, all had substance misuse issues or were under 18 years of age.

### Study selection

Following de-duplication, title and abstract screening was conducted by two reviewers (K.A. and J.L.), using CADIMA, an online evidence synthesis tool (Julius Kühn-Institut, Quedlinburg, Germany; https://www.cadima.info/index.php/area/evidenceSynthesisDatabase).^30^ Initially, 20% of titles and abstracts were screened independently and the remaining titles and abstracts screened once the inter-rater reliability score was substantial (0.61−0.80). Disagreements were resolved through discussion or by consultation with a wider team (L.P.G., J.A.S. and S.G.). Full-text screening was conducted independently by two reviewers (K.A. and J.L.), and disagreements were resolved with the same method.

### Data extraction and risk of bias

A standardised, pre-piloted form in Microsoft Excel for Windows (Microsoft Office 2019) was used to collate data about the intervention, comparison group, study design, sample size, country, demographics and outcomes for quality assessment and evidence synthesis. All outcome data with a comparison group were extracted as presented in the paper, making the unit of analysis clear. Data about both the total number of events and the number of participants experiencing an event were extracted. Two reviewers (K.A. and L.P.G.) extracted these data and resolved discrepancies through discussion, using consultation with wider team where necessary. The same two reviewers independently rated, for each extracted outcome, the seven categories of potential sources of bias for non-randomised studies in the Risk of Bias in Non-Randomized Studies – of Interventions (ROBINS-I) and the five categories of the Risk of Bias in Randomized Trials (RoB 2)^31^ for randomised controlled trials (RCTs). Discrepancies were resolved through discussion between reviewers, and the wider team if necessary. Each meta-analysis was then rated for the certainty of the evidence, using Cochrane's Grading of Recommendations, Assessment, Development and Evaluations (GRADE) framework.^32^ Certainty of the evidence was discussed for all reported outcomes, and noted in the paper where we considered the certainty to be very low (i.e. where the true effect is probably markedly different from the estimated effect).

### Data analysis

Results were synthesised by meta-analysis in Review Manager 5.4.1 for Windows (Cochrane Collaboration; https://training.cochrane.org/online-learning/core-software/revman/revman-5-download),^33^ supplemented by narrative synthesis where necessary. Where two or more studies reported outcomes suitable for pooling, meta-analyses were performed with random effects and 95% confidence intervals. Standardised mean difference models were used for continuous outcomes measured on a range of scales, and mean difference models were used for outcomes with a common scale (e.g. emergency department LOS measured in minutes). For events data reported per person, we used random effects relative risk (risk ratio) models with 95% confidence intervals where events were rare, and random effects odds ratios with 95% confidence intervals where events were relatively common (e.g. in-patient admissions), to make the association clearer.^34^ Analyses including studies assessed as at low or critical risk of bias were repeated in a sensitivity analysis excluding these studies, to check the sensitivity of the result to that study. Only unadjusted data were included in our meta-analyses. We assessed heterogeneity with the *I*^2^ statistic. Publication bias was to be checked with a funnel plot and the Egger test with Harbord modification^35^ in the case of categorical outcomes where there were at least ten studies in a meta-analysis (with fewer studies, the power of the test is too low).^36^

## Results

**Fig. 1:**
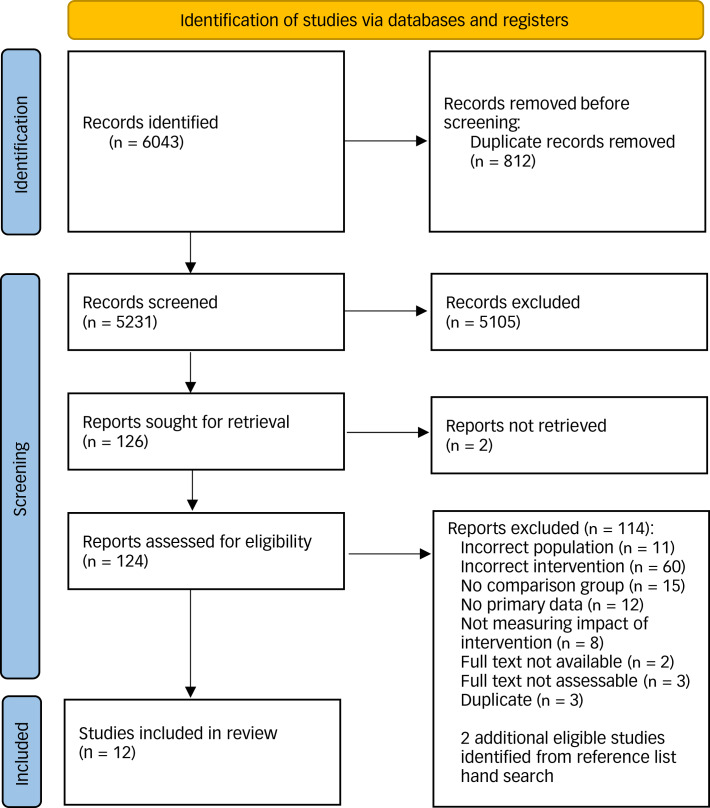
PRISMA flow diagram.

The search identified 6043 unique records, of which 124 were full-text screened and 12 met inclusion criteria (see Fig. 1). Of the 12 included studies, five were from the USA,^24,37–39^ three were from Australia^23,40,41^ one was from The Netherlands,^42^ Belgium,^43^ one was from the UK^25^ and one was from Canada.^44^ Methods included nine pre–post studies,^23,25,37–41,44^ one interrupted time series,^24^ one case–control study^43^ and one RCT,^42^ all written in English. Seven studies took emergency department patients as their population;^22,23,24,37,39–41^ in four studies, the population comprised patients referred or admitted to the unit.^38,42–44^ In one study, the population comprised people presenting via a mobile team (street triage).^25^ Across the studies, 67 505 participants were included (see Table 1).

**Table 1:** Characteristics of included studies

Study reference	Service description	Study design and duration	Setting	Participants (intervention)	Participants (comparison)	Participants, *N* (alternative *n*/comparison *n)*	Outcomes (primary outcome(s) in bold)
Braitberg et al, 2018^23^	24 h Behavioural assessment unit	Pre–post study 2-year comparison period	Australia, Royal Melbourne Hospital, 72 000 emergency department presentations per annum	Adults admitted to the behavioural assessment unit; no patients with a medical diagnosis	Adults aged ≥16 years, emergency department presentations with a length of stay between 3–24 h, diagnosis coded as a mental health issue, psychosocial crisis or related to intoxication	*n* = 5426 (2379/3047)	**Emergency department LOS,** time to emergency department clinician, time to emergency mental health clinician, emergency department security (‘code grey’) rates, emergency department restrictive intervention rates
Browne et al, 2011^40^	48 h psychiatric assessment and planning unit	Pre–post study, multiple comparison periods	Australia, Royal Melbourne Hospital	All emergency department patients, no transfers from other emergency departments or other psychiatric catchment areas	All emergency department patients, no transfers from other emergency departments or other psychiatric catchment areas	Not reported	Emergency department LOS, long waits in the emergency department, emergency department security (‘code grey’) rates, emergency department mechanical restraint rates, emergency department 1:1 nursing time, emergency department 1:1 nursing cost
Gillig et al, 1989^39^	24 h extended evaluation unit	Pre–post study, 2.5 week (intervention)/4-week comparison period; 30-day follow-up for intervention	USA, Louisville, Ohio (intervention); Cincinnati, Ohio (comparison). Ohio Valley urban area, 600 patient visits per month	Adults aged ≥18 years attending the psychiatric emergency service	Adults aged ≥18 years attending the psychiatric emergency service	*n* = 783 (435/348)	Hospital admission rates from emergency department, in-patient admissions (from emergency department and unit), hypothetical hospital admissions
Kealy-Bateman et al, 2019^41^	72 h joint short-stay unit and Missenden assessment unit	Pre–post study, 18-month (intervention)/unknown comparison period	Australia, Royal Prince Alfred Hospital, inner-city Sydney	Patients admitted to the short-stay unit via emergency department	Patients admitted to a psychiatric emergency care centre	Not reported	Admission to unit via emergency department
Lester et al, 2018^37^	48 h Crisis Assessment Linkage and Management (CALM) service	Pre–post study, 1-year comparison period, 30-day follow-up	USA, Ohio; 72 000 annual emergency department visits, ~7% for behavioural health complaints	Emergency department patients who received a psychiatric consult	Emergency department patients who received a psychiatric consult	*n =* 4598 (2387/2211)	**Emergency department LOS, hospital LOS, psychiatric hospital admission rate,** admission to unit via the emergency department, discharged from emergency department, 30-day readmission rate, unit LOS
Mok and Walker, 1995^44^	3-day short-stay unit	Pre–post study, 8-month comparison period	Canada, metro Halifax	Patients admitted to the regular stay unit	Patients admitted to regular stay unit	*n =* not reported (124/not reported),	Ward occupancy rates
Parwani et al, 2018^24^	24 h psychiatric observation unit	Pre–post study, ITS for analysis, 18-month comparison period	North Eastern USA; 1541-bed tertiary care academic medical centre	Adult emergency department patients aged ≥17 years evaluated by the acute psychiatry service. No patients who left the emergency department, were diverted elsewhere, never arrived or were discharged to court/law enforcement	Adult emergency department patients aged ≥17 years evaluated by the acute psychiatry service. No patients who left the emergency department, were diverted elsewhere, never arrived or were discharged to court/law enforcement	*n =* 7299 (3798/3501)	**Emergency department LOS, crisis intervention unit LOS, hospital LOS,** psychiatric hold rate, in-patient admission rate
Schneider and Ross, 1996^38^	3-day crisis unit	Pre–post study, 2-year comparison period 30-day follow-up	USA, Connecticut, community hospital	Patients admitted to the crisis unit	Patients admitted to the in-patient service	*n* = 1370 (590/780)	Average unit LOS, 30-day readmission rate
Spooren et al, 1997^43^	Three 72-h psychiatric crisis units	Pre–post study, case–control design, 6-month (pre)/13-month (post) comparison period, 1-month follow-up	Belgium, three urban hospitals (intervention); University of Ghent (comparison)	Emergency department patients referred every third week to the psychiatric crisis units	Emergency department patients referred every third week for short-term psychiatric in-patient treatment	*n* = 208 (171/37)	Psychological scales: symptoms. Patient reported improvement
Stamy et al, 2021^22^	Emergency psychiatric assessment, treatment, and healing unit (EmPATH)	Pre–post economic evaluation, 2-year comparison period	USA, Midwestern academic emergency department	All emergency department patients aged ≥18 years	All emergency department patients aged ≥18 years	*n* = 46567 (23231/23336)	**Emergency department revenue,** psychiatric boarding time (time waiting for a bed or for transfer), emergency department LOS, leaving emergency department without being seen, leaving emergency department against medical advice or eloped, in-patient admissions, fatalities
Trethewey et al, 2019^25^	Short-term psychiatric decisions unit	Pre–post study, 1-year comparison period	UK, Birmingham	All patients referred to the psychiatric decisions unit, emergency department presentations via street triage team and patients admitted to an in-patient unit following assessment by rapid assessment interface and discharge	All emergency department presentations via street triage team and patients admitted to an in-patient unit following assessment by rapid assessment interface and discharge	*n* = 980 (385/595)	In-patient admissions via liaison psychiatry, emergency department presentations via street triage
Van der Sande et al, 1997^42^	4-day special care unit for people who had attempted suicide (SOS-afdeling)	Randomised controlled trial, 12-month follow-up	The Netherlands, Utrecht University Hospital	Aged ≥15 years and attending for somatic treatment of the consequences of a suicide attempt	Aged ≥15 years and attending for somatic treatment of the consequences of a suicide attempt	*n* = 274 (140/134)	**Occurrence of subsequent suicide attempts,** medical care received in the year subsequent to index suicide attempt, psychological scales: symptoms, psychological scales: hopelessness

LOS, length of stay.

### Unit characteristics

Units could be designed to address multiple purposes. Five units were designed to reduce pressure on emergency department,^22,24,25,40,41^ four units were designed to provide a more therapeutic environment than the emergency department,^23,25,40,41^ three units were designed to reduce psychiatric admissions,^25,39,44^ three units were designed to reduce time spent in hospital^22,38,43^ and three units were designed to stabilise or improve patient well-being.^38,42,43^ Further purposes unique to a unit included to reduce the risk of future suicide attempts,^42^ reconnect with out-patient treatment,^38^ reduce out-of-area transfers^44^ and offer crisis-focused psychotherapy and case management services^37^ (see Table 2). Admission criteria were variable. Four units accepted patients likely to benefit from a short admission,^23,24,41,44^ and two units accepted people under the influence of drugs or alcohol.^23,41^ Units also specified acute behavioural disturbance,^23^ acute symptoms in relation to specific and short-term stressors,^38^ stable behaviour,^37^ requiring in-patient admission where there was no available bed^24^ or receiving medical treatment for a suicide attempt.^42^ Patients were excluded from admission if they were under the influence of or dependent on drugs or alcohol,^40,42^ displayed aggressive behaviour,^22,41^ had medical issues,^22,37,40,41^ resided outside of the catchment area,^40,42^ had a pattern of self-harming^42^ or required an in-patient admission.^22,42^

**Table 2:** Characteristics of units evaluated in included studies

Study reference	Unit	Maximum LOS	Capacity and location	Unit purpose	Further service details	Admission criteria	Referral pathway	Staffing
Braitberg et al, 2018^23^	Behavioural assessment unit	24 h	Six-bed unit	Move patients from the emergency department to a dedicated, well-resourced, low-stimulus environment	Fast-track the assessment and management of behaviourally disturbed patients presenting to the emergency department in an environment that has been specifically designed to be safe and secure, allow close observation and provide timely access to specialist expertise and facilities	Patients with acute behavioural disturbance, specifically behaviour influenced by drugs and alcohol, drug intoxication, mental illness and social crisis. Expected home discharge within 24 h	Emergency department	EMH and drug and alcohol clinicians. Two to three nurses always staffed the unit. A psychiatrist and/or psychiatry registrar every morning
Browne et al, 2011^40^	Psychiatric assessment and planning unit	48 h	Four-bed unit. Co-located within an expanded high-dependency unit in the Royal Melbourne Hospital Adult Acute In-patient Unit	Reduce emergency department mental health presentations and wait time for in-patient admissions. Improve care in a more appropriate, less restrictive environment	Intense management in the first 48 h of admission, including review of all new admissions by a consultant psychiatrist within 24 h to commence a clear management plan. Daily reviews by a consultant psychiatrist or psychiatry registrar then conducted	Patients from emergency department or within the catchment area requiring an admission for psychiatric evaluation and treatment. No transfers. Exclusions: intoxicated, sedated or requiring immediate medical attention	Emergency department or via local crisis assessment and treatment teams	Multidisciplinary team and allows patients and families to have access to psychiatric medical staff earlier in their episode of care than previously occurred, because of long lengths of stay in the emergency department waiting for admission previously
Gillig et al, 1989^39^	Extended evaluation unit	24 h	Not reported	Extended evaluation unit, or holding area, allowing up to 24 h of evaluation before making a referral. Purpose of which is to reduce hospital admission rates	No further information	Not reported	Psychiatric emergency service	Not reported
Kealy-Bateman et al, 2019^41^	Joint SSU and MAU	72 h	Six-bed unit. Located 300 m from the emergency department (SSU). Located adjacent to the SSU (MAU)	Appropriate and efficient care of patients who require brief admission and active therapeutic intervention before their return to community-based care (SSU). Attending to patients with a more specific health focus and in a timely manner, relieving pressure on the emergency department (MAU)	Developed in partnership with mental health and drug health services and the emergency department. Physically remote from the emergency department (approximately 300 m diagonally opposite) and able to provide some medical interventions, including intravenous therapy, because of staff with a mix of competencies	Patients with mental health problems likely to benefit from therapeutic intervention within 72 h, and patients with comorbid mental health, drug health or toxicology problems deemed suitable. Exclusions: aggression, acute medical or surgical problems, and more than two SSU admissions in 3 months (SSU). Not reported (MAU)	Emergency department, MAU, other parts of the hospital system (SSU). Emergency department or self-referral (MAU)	Initially a high proportion of emergency department staff with resuscitation skills, but this then reduced. Nursing:patient ratio of 1:2 and high levels of staff trained in trauma-informed care (SSU). Not reported (MAU)
Lester et al, 2018^37^	Crisis Assessment Linkage and Management (CALM) service	48 h	Eight-bed unit	Providing patients who would have boarded in the emergency department active behavioural treatment, e.g. crisis intervention focused psychotherapy, pharmacotherapy and case management services	Offers crisis intervention care delivered in a designated behavioural health unit located within the medical centre	Emergency department patients with psychiatric complaints and stable behaviour. No medical complaint. Other patients can be referred to the unit for observation care following initial psychiatric assessment in the emergency department	Emergency department	Psychiatric nurses and psychiatric care technicians in a 4:1 patient:staff ratio. Weekday coverage: an independently licensed social worker, a nurse practitioner and a supervising psychiatrist. Weekend coverage is provided by on-call psychiatry faculty
Mok and Walker, 1995^44^	SSU	3-day	Five-bed unit	Address decreasing in-patient beds and out-of-area transfers. Provide an integrated assessment, treatment and follow-up service facilitating discharge within 3 working days	All patients are advised about the brevity of admission and are encouraged to participate in treatment discharge planning. On discharge, patients have access to prompt follow-up through the out-patient department's Rapid Response Clinic, which holds out-patient clinics three times per week	Patients where discharge is likely within 3 working days, according to judgement of the assessing physician(s)	Primary referral route is the emergency physician, but any source accepted	A psychiatrist, psychiatry resident, staff nurse and medical social worker. After-hours nursing coverage is provided by staff from the regular-stay unit
Parwani et al, 2018^24^	Psychiatric observation unit	24 h	12-bed, locked unit	To reduce boarding and improve emergency department throughput of psychiatric patients in the emergency department	No further information	Any patient evaluated in the CIU, at the discretion of the CIU attending psychiatrist. Patients admitted were typically considered amenable to an observation stay less than 48 h or likely to require in-patient psychiatric admission but no bed was available	Via CIU, after psychiatric evaluation	Two nurses continuously as well as social workers and advanced practice providers during all days. Except for attending physician oversight often provided by the psychiatrist in the CIU, all staff are dedicated to the psychiatric observation unit
Schneider and Ross, 1996^38^	Crisis unit	3-day	Four-bed unit	Reduce time in hospital without reducing quality of care. Reduce acute symptoms, stabilise precipitating factors and reconnect with out-patient treatment	Treatment emphasises concrete problem-solving, education and medication stabilisation and adherence. Family therapy or other therapies, if relevant. Patients continually reminded that treatment is focused on crisis resolution and primary treatment site is the out-patient setting	Patients with acute symptoms attributable to specific and short-lived precipitants, such as medication non-adherence, disruption of important relationships and interruption of living arrangements	Not reported	0.25 FTE psychiatrist, 2.5 FTE registered nurses, 1 FTE psychiatric technician, 1 FTE crisis worker. Crisis unit and the traditional in-patient service share some minor responsibilities and clerical and administrative staff
Spooren et al, 1997^43^	Three psychiatric crisis units	72 h	Not reported	Stabilise condition of the patient. Improve well-being within a shorter time frame	Crisis management, consultations with partners and families, social interventions, short problem-focused therapy and motivational counselling toward further treatment. Some patients attended a limited follow-up to prepare them for further out-patient treatment	Not reported	Emergency department	Senior psychiatrist supported by psychiatric trainees and a multidisciplinary team of psychiatric and community nurses, social workers and a psychologist
Stamy et al, 2021^22^	Emergency psychiatric assessment, treatment and healing (EmPATH) unit	Not reported	12-person unit, recliners	Out-patient hospital- based program accepting emergency department patients in a psychiatric crisis. These units proclaim to decrease psychiatric boarding time and LOS at reduced costs compared with traditional psychiatric care	No further information	Patients in the emergency department considered appropriate. Must be nonviolent, not requiring in-patient psychiatry and medically cleared	Emergency department or via out-patient psychiatric clinic after consultation with an a unit psychiatrist	Psychiatrists, psychiatric nurses, nursing assistants, social workers and providers
Trethewey et al, 2019^25^	Psychiatric decisions unit	Not reported	Eight-person unit, no beds	Primary objective is to provide a safe, calm environment for enhanced assessment and short-term support to more complex patients in mental health crisis. Further aims are to relieve pressure on emergency department and avoid unnecessary in-patient admissions	No further information	Patients considered appropriate following an initial assessment by rapid assessment interface and discharge or street triage	Multiple: street triage team, rapid assessment interface and discharge teams (within the emergency department)	Not reported
Van der Sande et al, 1997^42^	Special care unit for people who had attempted suicide (SOS-afdeling)	4-day	Four-bed unit	Reduce the risk of further suicide attempts. Improve well-being	Brief in-patient treatment in a small, specialised psychiatric unit with subsequent 24 h emergency access to the unit, problem-solving out-patient treatment by a community nurse and home visits when necessary	Aged ≥15 years and receiving somatic treatment for the consequences of a suicide attempt. Exclusions: habitual self-harm, drug/alcohol problems, accidental overdose, inability to understand and write Dutch, residing outside the catchment area, psychiatric hospital admission, imprisonment, acute psychosis or recurrent consultations with a liaison psychiatrist during a prolonged stay (>2 days) on a somatic ward	Emergency department	One psychiatrist, two community psychiatric nurses, nine psychiatric nurses

LOS, length of stay; EMH, emergency mental health services; SSU, short-stay unit; MAU, Missenden assessment unit; CIU, crisis intervention unit; FTE, full-time equivalent.

Units received referrals from the emergency department,^22,23,25,37,40–44^ the psychiatric emergency service,^39^ other assessment and intervention units,^24,41,44^ out-patient clinics,^22,44^ other crisis services^25,40^ and other parts of the hospital.^22,41^ The units were most commonly staffed by psychiatrists,^23,24,37,38,40,42–44^ followed by social workers,^22,24,37,43,44^ nurses,^23,24,38,41,44^ psychiatric nurses,^22,37,42,43^ psychiatric technicians or nursing assistants,^22,38^ psychologists^43^ and drug and alcohol clinicians.^23^ Some units described themselves as hosting a multidisciplinary team^40,43^ or having a high staff/patient ratio, with high numbers of staff with knowledge of trauma-informed care.^44^

### Quality ratings

We extracted 41 outcomes from 11 non-randomised studies, and four outcomes from a single RCT. For the non-randomised studies, we assessed the majority of outcomes to have a moderate risk of bias (27 out of 41; see Supplementary Fig. 2). Risk of bias was typically limited because of strong study designs, which restricted both potential bias from confounding and potential bias from selection of participants. Potential bias in selection of reported result was the most common source of bias (in the absence of published protocols for most studies, it was unclear whether the full range of outcomes assessed had been reported). Three outcomes from two studies were at serious risk of bias from confounding, as there was reason to believe that the comparison and intervention groups were too dissimilar. Three further outcomes were at critical risk of bias from additional biases identified, or critical risk of bias from confounding. Seven outcomes from one study^22^ were assessed as having a low risk of bias. In the single RCT,^42^ potential bias in the randomisation process caused some concerns for two outcomes, and bias from missing outcome data caused two further outcomes to be of high concern for risk of bias (see Supplementary Fig. 3). The GRADE ratings are presented with the meta-analyses.

### Synthesis of outcomes

#### Emergency department waiting time

Three studies reported reductions in measures of waiting time in the emergency department. The first reported that the wait to be seen by a clinician in the emergency department was significantly reduced from a median of 68 min (interquartile range (IQR) = 24–130) in the control group to 40 min (IQR = 17–86) in the experimental group (*P* < 0.001).^23^ The same study also reported a significant median reduction in the wait time for a mental health review, from 139 (IQR = 57–262) to 117 (IQR = 49–224) min following the introduction of the crisis unit (*P* = 0.001).^23^ A further study reported that psychiatric boarding, the time waiting in the emergency department for a bed or transfer, was decreased from a median 212 (IQR = 119–536) to 152 (IQR = 86–307) min (mean difference 189 min, 95% CI 50–228 min).^22^ A third study reported a reduction in long waits in the emergency department. In the pre-period (between March 2006 and September 2006), there were at least 12 patients per month who waited in the emergency department for at least 24 h. In the post-period (January 2007 to January 2008), there were only six 24 h waits in the entire period (five of which were in the first month), and in the following 4 years only two patients waited in the emergency department for longer than 24 h.^40^

### Total LOS in the emergency department

**Fig. 2:**
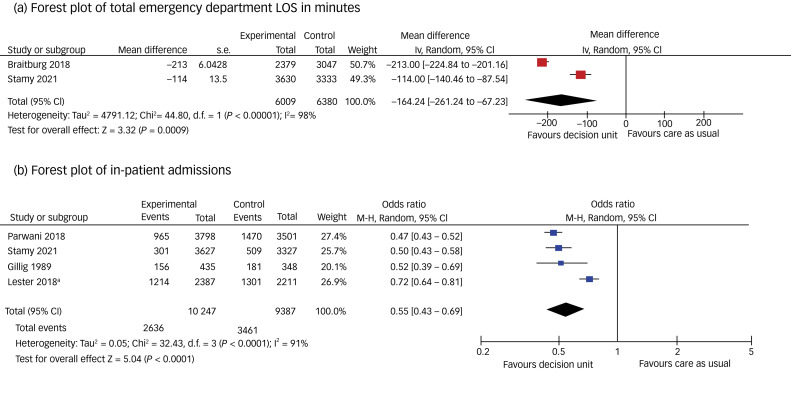
Forest plots for each meta-analysis. (a) Total emergency department LOS in minutes (decision unit vs care as usual), (b) in-patient admissions (decision unit vs care as usual). a. Admit + transfer. IV, inverse variance; LOS, length of stay; M–H, Mantel Haenszel.

A significant reduction in emergency department LOS was found by all four studies reporting this outcome.^22,23,24,37^ One study found a mean decrease from 14.48 to 11.11 h (significant *P* < 0.001) in a mixed-model analysis that used log-transformed emergency department LOS.^37^ Another study found a highly significant (*P* < 0.0001) change in the median emergency department LOS, from 155 (IQR = 19–346) to 35 (IQR = 9–209) min.^24^ Another study found a reduction in mean emergency department LOS from 423 (s.d. 265) min pre-intervention to 210 (s.d. 179) min post-intervention. Expressed as medians, this is a reduction from 328 (IQR = 227–534) to 180 (IQR = 101–237) min (*P* < 0.001).^23^ Another study found a significant reduction in median emergency department LOS from 351 (IQR = 204–631) to 334 (IQR = 212–517) min in the post-period, also expressed as a reduction in the mean of 114 min, with a 95% CI of 87–143 min.^22^ Data for the emergency department LOS were combined meta-analytically, using mean difference random-effects models (see Fig. 2(a)). The pooled estimate for change in total emergency department LOS is −164.24 min (95% CI −261.24 to −67.23 min; *P* < 0.001). Data from two studies could not be combined meta-analytically as one did not report a measure of variance^37^ and another only reported medians.^24^ The *I*^2^ is 98%, indicating high heterogeneity. The GRADE system assigns a starting rating of ‘Low certainty, confidence or quality’ to outcomes for meta-analyses of non-randomised studies. This was upgraded to ‘moderate certainty’ because of the ROBINS-I ratings, meaning that the authors believe that the true effect is probably close to the estimated effect.

### Leaving the emergency department early

One study^22^ reported no significant difference to the number of patients leaving emergency department without being seen, which was 2.4% of patients (*n* = 81) in the pre-period, and 2.2% (*n* = 79) in the post-period; a difference in proportions of −0.3 (95% CI −1.0 to 0.5). This study also reported no difference to the combined number of patients leaving against medical advice or eloped (meaning absconded or departed without authorisation). These were 2.4% (*n* = 81) in the pre-period and 2.0% (*n* = 74) in the post-period; a difference in proportions of −0.4 (95% CI −1.1 to 0.3).^22^

### Patient routes into emergency departments and wards

A UK study^25^ reported the number of patients who were brought to the emergency department via street triage (a mobile mental health service that works with the police, particularly on weekend evenings, to appropriately triage people displaying mental health problems away from the criminal justice system) in the pre- and post-periods. The number of patients brought to the emergency department via street triage reduced from 297 in the pre-period to 180 in the post-period (presumably as some patients were diverted to the crisis unit), but the authors did not test for significance. This study found that the number of patients admitted to a ward via liaison psychiatry reduced from 298 to 219 in the post-period, but did not test for significance.^25^ A further study assessed a crisis unit that allowed stays of up to 3 days at a site that already had a short-stay unit facilitating stays of up to 48 h.^41^ They report that 60% of patients admitted to the 3-day unit were admitted via emergency department, in contrast to the 48 h unit, for which almost all patients enter via emergency departments. The study was assessed as being at critical risk of bias, as it was unclear whether patients included in the control group would be equally eligible for the intervention group and *vice versa*.^41^

### The emergency department environment

Two studies reported changes in the use of security services and restraint procedures in the emergency department setting.^23,40^ Code grey events are formal team-led responses to risks to health and safety from actual or potential violent, aggressive, abusive or threatening behaviour from patients or visitors, directed internally or externally. One study reported a reduction in code grey events from 538 to 349 events (*P* = 0.003).^23^ These codes were called as a result of the behaviour of 370 patients in the pre-period compared with 259 patients in the post-period (*P* = 0.159).^23^ An additional study reported a reduction in code grey events in the emergency department from 101 to 88 per month (but this was accompanied by an increase in the number of planned code grey events from 10 to 30 per month for a linked unit).^40^

Examining restraint, all measures of restraint were reduced in both studies.^23,40^ The number of patients experiencing any restrictive intervention was reduced from 338 (12.7%) to 255 patients (10.7%) (*P* = 0.02).^23^ The number of physical restraints reduced from 339 (11.3%) to 224 events (9.4%) (*P* = 0.04).^23^ Similarly, there was a reduction in mechanical restraint from 275 (9.0%) to 156 events (6.6%) (*P* < 0.001).^23^ The use of therapeutic sedation was reduced from 250 (8.2%) to 156 (6.6%) (*P* < 0.001).^23^ The second study reported a 50% reduction in the total number of patients restrained, from 38 in the pre-period to 17 in the post-period, with an accompanying reduction in the total hours of restraint from 197 to 35 h.^40^ The average hours of mechanical restraint for individual patients also dropped from 6.8 to 2.5 h.^40^

### The ward environment

Only one study, which was assessed to be at moderate risk of bias,^44^ reported data for occupancy rates for the ‘regular stay unit’. These were 94%, 98%, 99% and 95% in the pre-period compared with 89% 91%, 96% and 85% in the post-period.^44^ The results are difficult to interpret, as the authors did not report the within-month variance or conduct a statistical analysis.

### Total time in crisis and acute care

One study reported changes to the total time in acute and crisis care (total time in the emergency department, crisis unit and in-patient admission). This was reduced from a mean of 100.89 (median 46.15) to 91.00 (median 31.35) h; a difference of around 10 h. Statistical testing used log-transformed data in a mixed model and found a significant reduction (*P* = 0.03).^37^

### Ward admissions and psychiatric holds

One study reported data about psychiatric holds, which can be voluntary or involuntary, are often 72 h in the US and are used for mental health evaluation.^24^ The psychiatric hold rate was significantly reduced (42% after the intervention compared with 49.8% before the intervention; difference of 7.8%; *P* < 0.0001).

All four studies reporting ward admissions reported a significant reduction. In one study, this was reduced from 42% to 25% of patients (*P* < 0.001).^24^ A further study reported 301 (8.3%) of patients presenting to emergency department were admitted to in-patient psychiatry in the post-period compared with 509 (15.3%) patients in the pre-period, representing a significant difference of −7.0% (95% CI −8.5 to −5.5).^22^ A third study^37^ reported that fewer patients were either admitted to a ward or discharged from the emergency department. Admissions reduced from 47.9% of presenting psychiatric patients to 38.0%. Rates of discharge from emergency department were also reduced from 39.1% to 28.2%. Transfers remained similar. The total number of admissions to ward and transfers reduced from 58.8% to 50.9%.^37^ The fourth study reported that 35% (156/435) of the intervention group were admitted to hospital from either the emergency department or the crisis unit (10% (42/435) of these were admitted from the emergency department), compared with 52% (181/348) of the control group,^39^ but these outcomes were assessed as being at serious risk of bias because of the differences between the sites and populations they serve.

The data for ward admissions from these four studies were combined meta-analytically, and a sensitivity analysis was conducted excluding the study at critical risk of bias^39^ (see Fig. 2(b) for meta-analysis and Supplementary Fig. 4 for the forest plot of the sensitivity analysis). The combined odds ratio is 0.55 (95% CI 0.43–0.68), with an *I*^2^ value of 91% (data from 19 634 patients). The result was effectively unchanged in the sensitivity analysis (combined odds ratio of 0.55, 95% CI 0.42–0.73, *I*^2^ of 94%). The GRADE rating was upgraded to ‘moderate certainty’, meaning that the authors believe that the true effect is probably close to the estimated effect.

### Hospital admissions in follow-up

Two out of three studies reported no difference to hospital admissions in the follow-up period. The rate of hospital admissions across the groups over a 1-month follow-up period was 6.9% compared with 6.7% in one study.^37^ A further study, reporting on a unit specialising in helping people who attempted suicide, recorded a reduction in the number of patients who had a psychiatric in-patient admission during a 1-year follow-up in the crisis unit group (24%) compared with (38%) participants in the comparison group, but did not test for significance.^42^ A final study reported that the 30-day readmission rate for those who stayed in the intervention unit was similar to other patient samples, but this was deemed to be at serious risk of bias as the comparison group(s) were not clearly defined.^38^

Two studies reported LOS for hospital admissions. The first study reported no significant difference between the number of in-patient days when the time spent on the experimental unit was included: 33 (s.d. 73.5) days in the active group and 37 (s.d. 83.0) days in the control group.^42^ However, when the in-patient days were compared excluding the time on the experimental unit, the difference was significant (*z* = −5.51, *P* < 0.001).^42^ The second study reported the modal LOS for this outcome: in the pre-period, only those admitted to a ward were included (modal LOS of 5 days).^37^ However, it should be noted that many of the stays in the follow-up seemed to be very short visits to the crisis unit, which is driving the modal LOS in the post-period to be 1 day. This introduces bias as the admission criteria for the two groups (stay on crisis unit versus stay on ward) is not comparable.

### Clinical and patient experience

There we no significant difference between the groups for clinical and patient experience outcomes. The first study compared scores on the General Health Questionnaire (GHQ-28)^45^ between groups at follow-up and found no significant difference (*t* = −0.37, *P* = 0.715) (moderate risk of bias).^43^ This study also collected data for the patient reported improvement, and found no difference between the proportions of patients who reported improvement in each group (*t* = 0.42, *P* = 0.677).^43^ The second study reported no significant effect of treatment on either the General Symptom Index (*F*(8,112) < 1, *P* = 0.72),^42^ Hopelessness Scale (*F*(1,110) = 2.14, *P* = 0.15)^42^ or Symptom Checklist (SCL-90) (*F*(8,110) = 1.03, *P* = 0.42).^42^

### Suicidality

One study described an experimental unit designed for those who had attempted suicide, and is the only study that reported data about changes to suicidality.^42^ The study concluded that the unit had no impact on the frequency of suicide attempts compared with treatment as usual. There was no significant difference in the probability of repeat suicide attempts in the follow-up period (hazard ratio of repetition for patients in the experimental group compared with the care as usual group was 1.24; 95% CI 0.68–2.27). Congruently, there was no difference in the number of suicide attempts per patient in the follow-up period (*z* = 0.49, *P* = 0.62).^42^ Patients at high risk of a repeat suicide attempt can be defined by a score of at least four on the Buglass and Horton (1974) Scale.^46^ Using only these patients, a non-significant difference in repeat attempts was found between the experimental and control groups (log rank test = 2.69, *P* = 0.10).^42^

### Follow-up out-patient care

Significantly more patients in the experimental group received out-patient care (including care specifically connected to the unit) in the first year of follow-up (χ^2^ = 37.42, d.f. = 1, *P* < 0.001).^42^

### Fatalities

One study reported data for any fatalities of study participants.^22^ In the ‘pre’ sample of 3333 emergency department psychiatric presentations, there was one fatality; and in the ‘post’ sample of 3630 emergency department psychiatric presentations, there were no fatalities. This outcome was not sufficiently powered for conclusions to be drawn, and suicidality was not a specific inclusion or exclusion criteria for the study.

### Health economics outcomes

Two studies reported health economics outcomes. One reported a reduction in time spent in the emergency department for those presenting with psychiatric problems, and a congruent decrease in the number of hours (by 1475 h) of one-to-one nursing care in the emergency department in the first 3 months after the unit fully opened compared with the same period in the previous year, translating to an annual reduction in the cost of one-to-one nursing in the emergency department of $120 088 (international dollars).^40^ However, neither a denominator or significance test was reported. The second reported additional revenue for the emergency department resulting from the short-stay crisis unit opening of $404 954 (USD) in the initial 6 months and $861 065 annually.^22^

## Discussion

### Main findings

This is the first systematic review of short-stay crisis units for mental health patients on the crisis care pathway. Units typically have two service-defined objectives: to reduce waiting time in the emergency department and/or to reduce in-patient admissions. Our review is indicative of a significant reduction in both outcomes. Mental health crisis services have been described as being themselves in crisis, experiencing pressure from busy emergency departments and wards operating at or beyond capacity, including during the COVID-19 pandemic.^1,3–5,7,8^ As such, these findings are of interest and relevance to many stakeholders; those designing and commissioning services, service providers, clinicians, patients, carers and bodies assessing the quality of services (e.g. the Care Quality Commission in the UK). Reducing time spent in the emergency department by using short-stay crisis units as part of the crisis care pathway^23,24,37,22^ could help to improve the flow of patients through the emergency department,^20,21^ and so these findings are potentially important to general hospitals struggling with emergency department capacity and planning.^2,3^ We also found that the likelihood of an in-patient admission for people in crisis was reduced following a stay on a crisis unit (compared with patients not accessing crisis units).^24,37,22,39^ It is possible that crisis units are functioning to delay in-patient admission,^38^ although in one study the total time spent in crisis and acute care following the index crisis presentation was reduced.^37^ There was also a suggestion that short-stay crisis units offer more time to make the most appropriate decision about admission, discharge or community referral when the best course of action is unclear,^37^ indicating the potential for the risk of inappropriate in-patient admissions^17^ or premature emergency department discharges to be decreased.

For patients and their carers it may be more important that the unit provides a better patient experience than the chaotic environment of the emergency department,^9^ offering an alternative space for stabilisation of crisis and assessment. Four studies included in the review reported providing a more therapeutic environment as a stated aim of the unit.^23,25,40,41^ A key measure related to creating a more therapeutic experience is the use of restrictive interventions,^9,47^ with two studies finding a reduction in the use of security and restraint services in the emergency department^23,40^ (although in one of those studies the decrease in security events in the emergency department occurred alongside an increase in security events elsewhere on the pathway).^40^ In addition, short-stay crisis units do not seem to have significant effects on mental health outcomes, as measured by standardised scales assessing distress, symptoms and hopelessness.^42,43^ Two studies assessed the patient experience directly, finding no difference in outcome,^25,43^ and no social outcomes were included in any study. As such, we note that data on the patient experience of short-stay crisis units were limited, and it was not possible to draw conclusions on whether crisis units improved the overall patient experience of the crisis care pathway.

Finally, many people presenting in crisis at emergency department are feeling suicidal,^48^ and just one study reported outcomes related to suicidality, finding that staying on a short-stay crisis unit did not change the probability or number of repeat suicide attempts.^42^ Given that managing the risk of harm to self and others is a key focus of mental health crisis care, it is of note that the existing literature does not offer better evidence of the effect of crisis units on suicidality.

### Limitations

Where we were able to undertake meta-analyses, we felt that the quality of studies indicated moderate certainty that our estimates of effect were close to the true value, and as such suggest that our findings are of relevance to stakeholders in mental health crisis care. However, we do recognise that differences in short-stay crisis units’ operational structure and issues around capacity elsewhere on the crisis care pathway (e.g. in-patient beds) at a local level are likely to mediate any effect that can be expected of crisis units. Studies reported many outcomes of interest, with data synthesis indicating wide potential benefits of crisis units. However, differences between studies in the way outcomes were reported (for example, reporting median rather than mean values) limited the number of meta-analyses we could perform, and therefore the range of conclusions we were able to draw from the evidence. We were unable to adequately explore the effects of crisis units on patient experience and suicidality, highlighted by researchers on the team working from a lived experience perspective as being of particular importance, limiting the scope of our review. Similarly, there was insufficient evidence reported to properly consider the health economic effects of crisis care units.

### Clinical implications

There is evidence to suggest that short-stay crisis units can address service priorities effectively (reducing demand on the emergency department and psychiatric in-patient facilities, decreasing psychiatric hold rates and increasing rates of out-patient follow-up), suggesting that these units offer a useful addition to the crisis care pathway. We note that units tended to focus on either improving outcomes in the emergency department or on psychiatric admissions, and it may be more difficult to configure units in such a way to effectively address both service pressures. When establishing crisis units, it may be sensible to consider the priorities of the particular healthcare context that drive the initiation of these units when thinking about their utility in local services (e.g. in addressing the financial burden of extended emergency department boarding, or lack of in-patient capacity). Given our lack of findings related to patient experience and suicidality, it is also important to consider potential harms of introducing crisis units (e.g. fragmentation of the pathway as a series of very short stays in different facilities).

### Future research

As noted above, future research is needed to improve the evidence base for the impact of short-stay crisis units on patient experience, suicidality, and clinical and social outcomes, including longer-term effects. Higher-quality studies, including RCTs, would strengthen confidence in the evidence. Research is needed to investigate the relationship between a unit's aim(s), configuration and outcome(s). This includes system-level studies that examine the role of short-stay units as part of a crisis care pathway, including more research on health economic costs and benefits at both unit and system level.

In conclusion, there is good evidence that short-stay crisis units, provided for people on a mental health crisis care pathway, can achieve the primary goals of reducing pressure on the emergency department and in-patient admissions, and secondary goals of decreasing psychiatric hold rates and increasing out-patient follow-up rates. This is encouraging for service providers who are struggling to manage the flow of patients from emergency department and along the crisis care pathway, and for patients who are not best served by an unhelpful admission and who would benefit from follow-up care. Although the stated purpose of units is also to provide a more therapeutic environment than the emergency department and to improve patient experience, there is limited evidence to suggest the units accomplish these aims. Further research is needed to identify the effects on the patient experience and to ensure that crisis units are best configured to meet the needs of the healthcare system locally.

## Data Availability

The data that support the findings of this study are available from the corresponding author, L.P.G., upon reasonable request.

## Supplementary material

For supplementary material accompanying this paper visit http://doi.org/10.1192/bjo.2022.534. click here to view supplementary material
