# Supplementary material for: Short-stay crisis units for mental health patients on crisis care pathways: systematic review and meta-analysis
Source: BJPsych Open. 2022 Jul 25;8(4):e144. doi: 10.1192/bjo.2022.534 (PMC9344431; doi:10.1192/bjo.2022.534)
Supplement: Supplementary file 1 [file S2056472422005348sup001.docx]

## Online Supplementary Materials:

**Online Supplement Figure 1.** Database search strategy

**MEDLINE (OVID)**

1.((mental* or psycho* or psychiatr*) adj3 (emergenc* or crisis or crises)).mp.

2.mental disorders/ or personality disorders/ or "schizophrenia spectrum and other psychotic disorders"/

3. Preventive Psychiatry/ or Biological Psychiatry/ or Community Psychiatry/

4. problem behavior/ or self-injurious behavior/ or self mutilation/ or suicide/

5. Social Behavior Disorders/

6.(triage adj3 (ward* or unit*)).mp

7.Emergency Service, Hospital/ or Emergency Services, Psychiatric/

8. ((asses* or evaluat* or stabilis* or stabiliz* or crisis or crises or observational*) adj4 (unit or units or facilit* or ward* or room* or suite* or service*)).mp.

9.hospital units/ or clinical observation units/

10."psychiatric intensive care unit*".mp.

11."psychiatric emergency service*"

12."alameda model".mp.

13.psychiatric emergency department.mp

14."Short-stay unit".kw. 15.23 hour.mp.

16.crisis intervention/

17.Psychiatric Department, Hospital/

18. "psychiatric emergency care centre*".mp.

19.exp clinical study/

20.((randomi* or control* or "double-blind") adj3 (trial* or stud* or group* or experiment* or design* or investigation* or procedure* or intervention*)).mp.

21."interrupted time series".mp.

22."two group stud*".mp.

23."cohort ".mp

24.(before adj3 after).mp.

25.(pre adj3 post).mp.

26."comparison group*".

27.(quasi adj2 experimental*).mp.

28.Comparative Study/

29.retrospective*.mp.

30.longitudinal*.mp.

31.exp Cohort Studies/

32. 1 or 2 or 3 or 4 or 5

33. 6 or 7 or 8 or 9 or 10 or 11 or 12 or 13 or 14 or 15 or 16 or 17 or 18

34. 19 or 20 or 21 or 22 or 23 or 24 or 25 or 26 or 27 or 28 or 29 or 30 or 31

35. 32 and 33 and 34

**PSYCINFO (OVID)**

1.((mental* or psycho* or psychiatr*) adj3 (emergenc* or crisis or crises)).m

2.mental disorders/ or affective disorders/ or borderline states/ or personality disorders/ or "stress and trauma related disorders"/

3.Psychiatric Patients/

4.Behavior Disorders/

5.Suicide Prevention/ or Suicidal Ideation/ or Self-Injurious Behavior/ or Distress/

6.psychiatric symptoms/

7.(triage adj3 (ward* or unit*)).mp

8.crisis intervention/ or crisis intervention services/ or emergency management/

9.psychiatric units/

10.((asses* or evaluat* or stabilis* or stabiliz* or crisis or crises or observational*) adj4 (unit or units or facilit* or ward* or room* or suite* or service*)).mp

11."psychiatric intensive care unit*".mp.

12."mental health and illness assessment"/

13.triage unit.mp.

14."psychiatric emergency service*".mp

15.psychiatric emergency department.mp.

16."Short-stay unit".mp.

17.Crisis Intervention/

18."psychiatric emergency care centre*".mp.

19.clinical trials/ or randomized controlled trials/

20.Cohort Analysis/

21.quantitative methods/

22.exp experimental design/

23.((randomi* or control* or "double-blind") adj3 (trial* or stud* or group* or experiment* or design* or investigation* or procedure* or intervention*)).mp

24."interrupted time series".mp.

25."two group stud*".mp.

26."cohort".mp.

27.(before adj3 after).mp.

28.(pre adj3 post).mp.

29."comparison group*".mp

30.(quasi adj2 experimental*).mp.

31.retrospective studies/

32.retrospective*.mp.

33.Longitudinal Studies/

34.cohort stud*.mp.

35.Improvement. mp

36. 1 or 2 or 3 or 4 or 5 or 6

37. 7 or 8 or 9 or 10 or 11 or 12 or 13 or 14 or 15 or 16 or 17 or 18

38. 19 or 20 or 21 or 22 or 23 or 24 or 25 or 26 or 27 or 28 or 29 or 30 or 31 or 32 or 33 or 34 or 35

39. 36 and 37 and 38

**EMBASE (OVID)**

1. ((mental* or psycho* or psychiatr*) adj3 (emergenc* or crisis or crises)).mp.

2. anxiety disorder/ or behavior disorder/ or emotional disorder/ or mood disorder/ or personality disorder/ or psychosis/ or schizophrenia spectrum disorder/

3. emergency psychiatry/ or liaison psychiatry/

4. behavior disorder/

5. (triage adj3 (ward* or unit*)).mp.

6. psychiatric emergency service/

7. ((asses* or evaluat* or stabilis* or stabiliz* or crisis or crises or observational*) adj4 (unit* or units or facilit* or ward* or room* or suite* or service*)).mp.

8. observation unit/

9. "psychiatric intensive care unit".mp.

10. "psychiatric emergency service*".mp.

11. "alameda model".mp.

12. psychiatric emergency department.mp.

13. "short-stay unit".mp.

14. "23-hour".mp.

15. crisis intervention/

16. ((randomi* or control* or "double-blind") adj3 (trial* or stud* or group* or experiment* or design* or investigation* or procedure* or intervention*)).mp.

17. clinical study/

18. exp epidemiology/

19. cohort stud*.mp.

20. (before adj3 after).mp.

21. "interrupted time series".mp.

22. "two group stud*".mp.

23. (pre adj3 post).mp.

24. (before adj3 after).mp

25. exp controlled study/

26. intermethod comparison/

27. comparative study.mp.

28. retrospective*.mp.

29. longitudinal*.mp

30. exp follow up/

31. 1 or 2 or 3 or 4

32. 5 or 6 or 7 or 8 or 9 or 10 or 11 or 12 or 13 or 14 or 15

33. 16 or 17 or 18 or 19 or 20 or 21 or 22 or 23 or 24 or 25 or 26 or 27 or 28 or 29 or 30

34. 31 and 32 and 33

**CINAHL (EBSCO)**

S1 (mental* or psycho* or psychiatr*) N3 (emergenc* or crisis or crises)

S2 (asses* or evaluat* or stabilis* or stabiliz*) N4 (unit* or facilit* or ward* or room* or suite* or service*)

S3 (MH "Personality Disorders") OR (MH "Psychotic Disorders") OR (MH "Mental Disorders")

S4 (MH "Schizophrenia")

S5 (MH "Psychiatric Emergencies") OR (MH "Psychiatry") OR (MH "Psychiatric Units") OR (MH "Psychiatric Service") OR (MH "Psychiatric Patients")

S6 (MH "Hospital Units")

S7 (MH "Observation Units")

S8 (MH "Disruptive Behavior") OR (MH "Social Behavior Disorders")

S9 (MH "Suicide, Attempted") OR (MH "Suicidal Ideation") OR (MH "Suicide") OR (MH "Suicide Risk (Saba CCC)")

S10 (MH "Suicide Prevention (Iowa NIC)")

S11 "psychiatric intensive care unit*"

S12 "clinical stud*"

S13 (randomi* or control* or "double-blind") N3 (trial* OR stud* OR group* OR experiment* OR design* OR investigation* OR procedure* OR intervention*)

S14 "interrupted time series"

S15 "two group stud*"

S16 "cohort stud*"

S17 before N3 after

S18 pre N3 post

S19 "behavioural assessment unit*" OR "behavioral assessment unit*"

S20 "psychiatric emergency service*"

S21 "alameda model"

S22 "crisis stabilization unit*" OR "crisis stabilisation unit*"

S23 "triage ward*"

S24 assess* or evaluat* or stabilis* or stabiliz*

S25 S1 OR S3 OR S4 OR S8 OR S9

S26 S2 OR S6 OR S7 OR S11 OR S19 OR S20 OR S21 OR S22 OR S23

S27 S12 OR S13 OR S14 OR S15 OR S16 OR S17 OR S18

S28 S24 AND S25 AND S26 AND 27

S29 (MH “Prospective Studies”)

S30 (MH “Retrospective Design”)

S31 S27 OR S29 OR S30

S32 S25 AND S26 AND S31


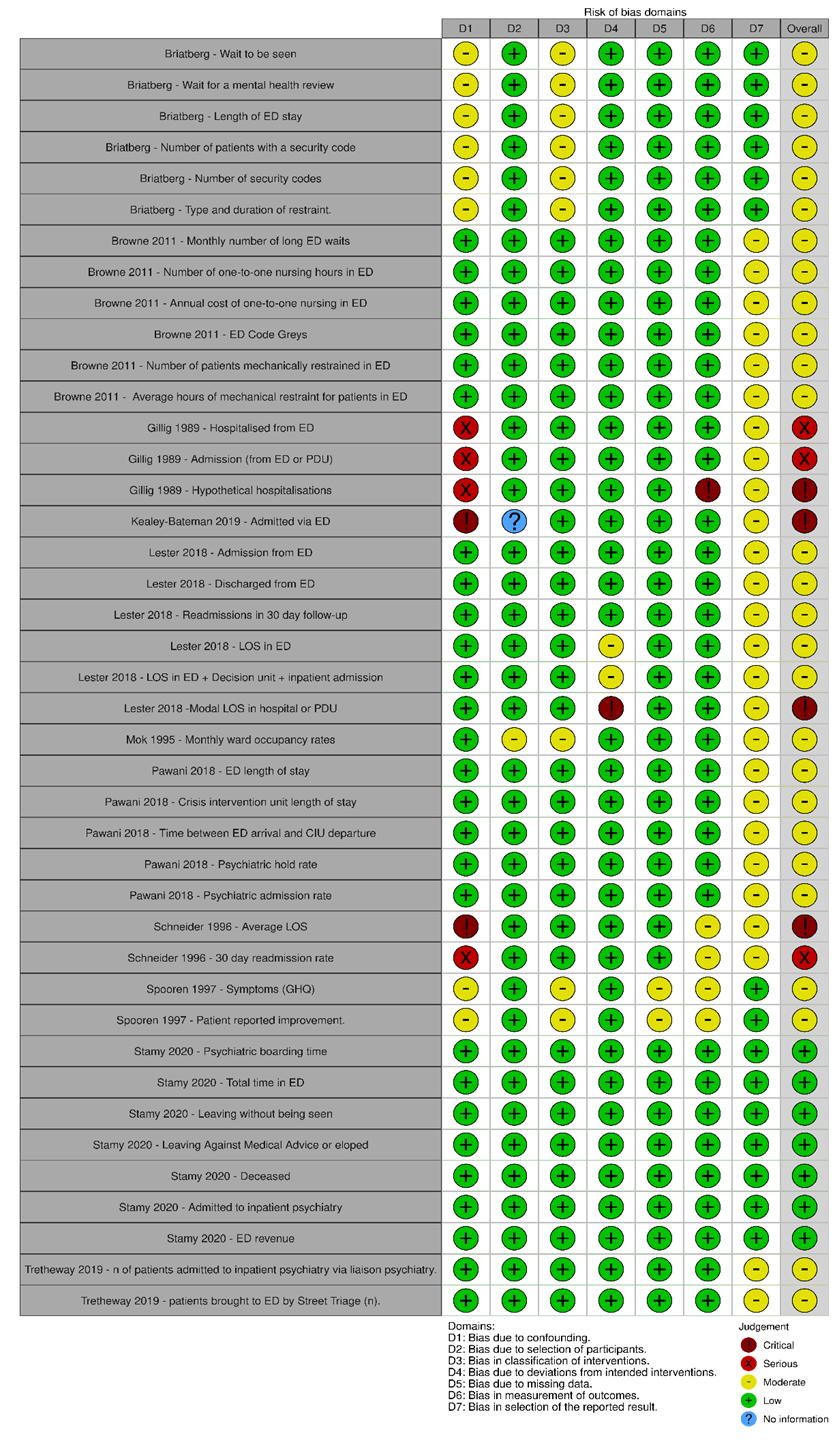
**Online Supplement Figure 2.** ROBINS-I quality rating for outcomes from the non-randomised studies

**Online Supplement Figure 3.** Risk of Bias (ROB 2) ratings for outcomes for the RCT (Van der Sande 1997)


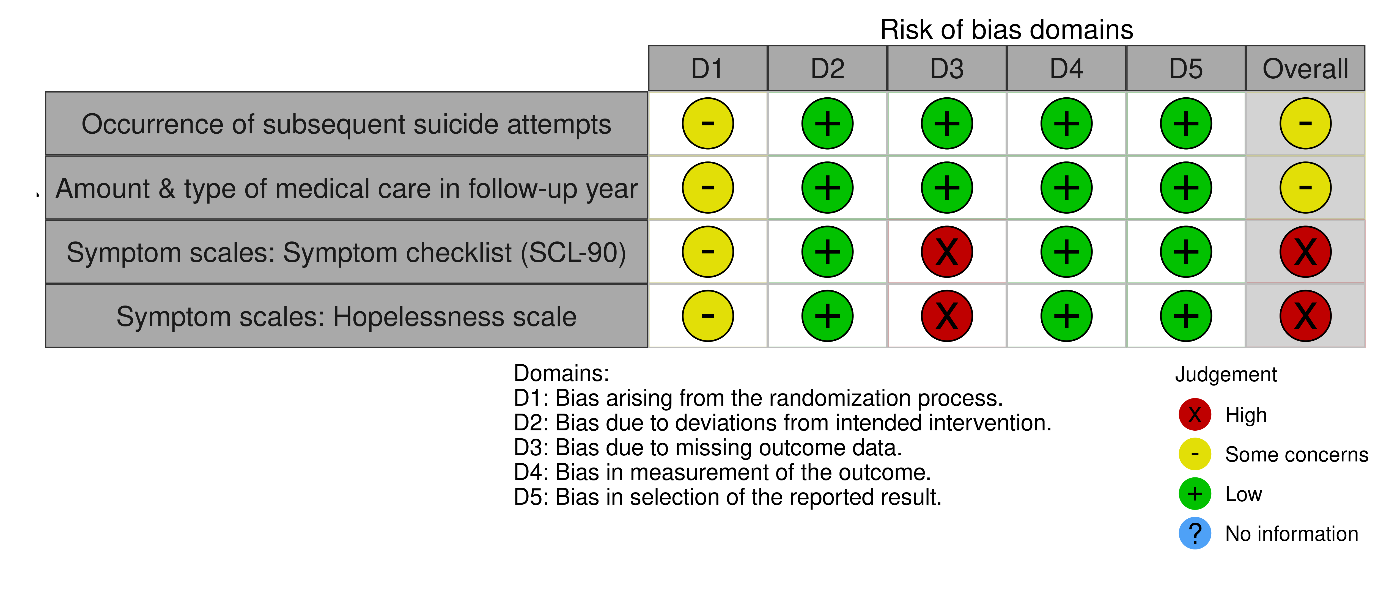


**
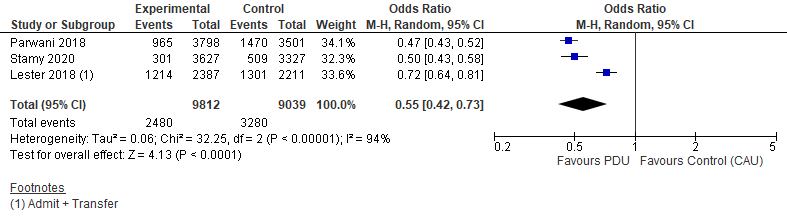
Online Supplement Figure 4.** Sensitivity analysis of meta-analysis of Inpatient Admissions
